# Predictors of Diabetic Foot Reulceration beneath the Hallux

**DOI:** 10.1155/2019/9038171

**Published:** 2019-01-08

**Authors:** R. J. Molines-Barroso, J. L. Lázaro-Martínez, J. V. Beneit-Montesinos, F. J. Álvaro-Afonso, E. García-Morales, Y. García-Álvarez

**Affiliations:** Diabetic Foot Unit, Facultad de Medicina, Universidad Complutense de Madrid, Instituto de Investigación Sanitaria del Hospital Clínico San Carlos (IdISSC), Madrid, Spain

## Abstract

**Aims:**

To evaluate the factors that predict reulceration beneath the hallux in people with a history of diabetic foot ulceration.

**Methods:**

A prospective study conducted between January 2012 and December 2014 was performed in a diabetic foot unit to assess the risk factors associated with hallux reulceration. Sixty patients with diabetic neuropathy and a history of previous ulcer were consecutively included. Sociodemographic factors and comorbidities plus the biomechanical and radiographic factors were obtained. Follow-up on participants was conducted every month, and they wore offloading therapeutic footwear and custom-made insoles. Hallux reulceration during the follow-up period was assessed as the main outcome measure in the study.

**Results:**

Patients were followed up during 29 (14.2-64.4) months. Twenty-nine patients (52%) developed a new ulceration: 9 patients (31%) in the hallux and 20 (69%) in other locations. Functional hallux limitus (*p* = 0.005, 95% CI (2.097–73.128), HR 12.384) and increased body mass index (*p* = 0.044, 95% CI (1.003-1.272), HR 1.129) were associated with the hallux ulceration-free survival time in the multivariate Cox model.

**Conclusions:**

Obesity and the presence of functional hallux limitus increase the probability of developing hallux reulceration in patients with diabetic neuropathy and a history of ulcers.

## 1. Introduction

The lifetime incidence of foot ulcers in people with diabetes has been recently estimated to be between 19% and 34% [1]. At least 85% of lower-extremity amputations are preceded by a diabetic foot ulcer (DFU), which severely increases the economic costs of health care and decreases life expectancy [2]. The primary prevention of DFU becomes critical, since death in the first year following diagnosis of the first DFU has been reported in as many as 12% of patients [3].

Diabetic neuropathy, preulcerative lesion, peripheral arterial disease, foot deformity, and increased plantar pressure have been identified as the main risk factors for DFU [4, 5]. Forty percent of patients will have a recurrence within 1 year following healing of the ulcer. The precipitating factors that initially led to the ulcer are generally not resolved after healing [1].

The forefoot is the area of higher prevalence of DFU. In particular, the hallux constitutes one-third of all areas affected by DFUs [6]. Hallux reulceration can lead to hallux amputation which has devastating effects on foot biomechanics and increases the risk of new ulcers and lower-extremity amputation [7]. Research on the risk factors of hallux reulceration can lead to the reduction of the incidence of ulcers and help avoid hallux amputation and its devastating consequences.

The normal range of motion (ROM) of the first metatarsophalangeal joint (first MTPJ) is defined as being more than 65° dorsiflexion, while hallux rigidus is the severe limitation of hallux dorsiflexion (<30°) [8, 9]. The ROM of the first MTPJ is routinely evaluated in a non-weight-bearing position in patients with diabetes at high risk of ulcer [10, 11]. However, mobility of the first MTPJ in the resting position has proven to be a poor predictor of abnormal first ray function during gait [12]. Foot examination in a non-weight-bearing position can be normal, but dorsiflexion of the first MTPJ can be blocked during gait, which means that this risk factor may remain unrecognized. This condition whereby range of motion is reduced when the forefoot is loaded is referred to as functional hallux limitus [13].

Research conducted by Nubé et al. [14] and Cowley et al. [15] evaluated the limited mobility of the first MTPJ in relation to ulcer location on the hallux, but they were unable to find an association. ElMakki et al. [16] reported a relationship between hallux ulceration and a group of deformities which included limited mobility of the first MTPJ. These research studies [14–16] evaluated the factors associated with hallux ulcers in patients with diabetes and measured the range of motion of the first MTPJ in the resting position.

Boffeli et al. [9] explored the ROM of the first MTPJ both in the resting position and in the weight-bearing position in a group of patients with hallux ulceration. These authors [9] demonstrated that almost all patients exhibited limited first MTPJ mobility, and almost half of the patients presented functional hallux limitus.

A higher prevalence of limited mobility of the first MTPJ has been reported in patients with previous hallux ulceration [9]; however, to date, the association between hallux reulceration and limited mobility of the first MTPJ has not yet been explored by means of a prospective follow-up study.

Our study aim was to evaluate the factors that predict reulceration beneath the hallux in people with a history of diabetic foot ulceration.

## 2. Materials and Methods

A prospective study was conducted between January 2012 and December 2014 involving patients admitted to a diabetic foot unit at the Complutense University of Madrid in Spain, which is an outpatient center. Sixty individuals were consecutively evaluated according to the following criteria: aged over 18 years, diagnosed with type 1 or type 2 diabetes mellitus (DM) according to the criteria of the American Diabetes Association, presence of peripheral neuropathy, presence of a first event of a recently healed ulcer, and location of the ulcer on the forefoot. Baseline clinical data are shown in Table 1.

Patients who met the following criteria were excluded: those with peripheral arterial disease (PAD), active ulcer, diabetic neuropathic osteoarthropathy, history of previous amputation, ulcer caused by trauma, history of rheumatoid arthritis, or disease causing peripheral neuropathy other than DM.

PAD was considered when both distal pulses were absent and/or the ankle brachial index (ABI) was <0.9. In patients whose ABI was >1.4 or in those with diagnostic uncertainty, a toe pressure of <55 mmHg or a toe brachial index of <0.7 was used to diagnose PAD [17].

The local ethics committee approved this study, and all patients signed their informed written consent, in accordance with the principles of the Declaration of Helsinki.

### 2.1. Peripheral Sensory Neuropathy

Neuropathy was diagnosed by using the Semmes-Weinstein 5.07/10 g monofilament and a biothesiometer (both from Novalab Ibérica, Madrid, Spain) [18]. The presence of peripheral sensory neuropathy was confirmed by a lack of feeling detected in either one or both of the tests.

### 2.2. Biomechanical Assessment

Foot type was classified using the validated protocol of the Foot Posture Index (FPI-6) [19]. A total FPI-6 score between 0 and +5 indicates a neutral foot, a score of above +6 indicates a pronated or highly pronated foot, and a score between -1 and -12 indicates a supinated or highly supinated foot.

Hallux deformities were considered when the hallux presented one of the following: hallux valgus, bony prominence of the first metatarsal head, or hallux hammertoe deformity [7, 9, 20].

The range of mobility of the following joints was measured by using a two-armed goniometer: the ankle joint, the subtalar joint, and the first MTPJ.

Ankle dorsiflexion was examined with the patient in the supine position, keeping the subtalar joint position neutral while forcefully dorsiflexing at the ankle joint and measuring the angle formed between the bisections of the fibula and lateral foot, which had been previously marked on the patient's skin [20].

The ROM of the subtalar joint was examined with the patient in the prone position and holding the calcaneus with one hand and the talar head/neck with the thumb and index finger of the other hand. The adduction (inversion) and abduction (eversion) ROM were assessed with the hand on the calcaneus [20].

**Figure 1:**
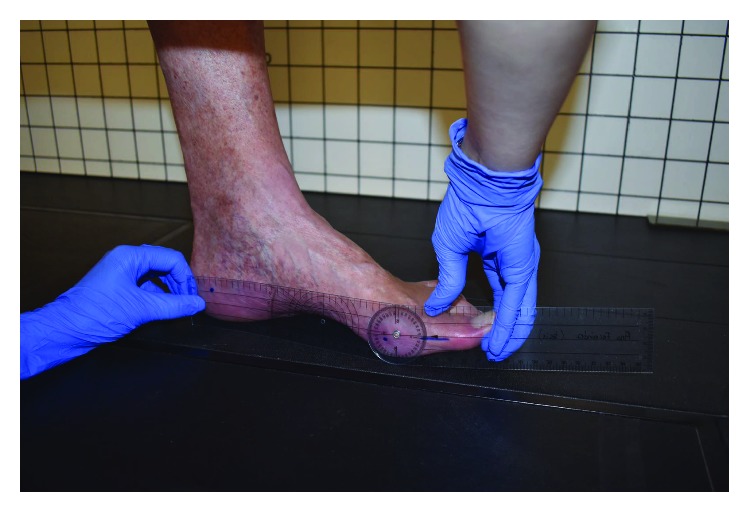
Evaluation of the first MTPJ dorsiflexion in a weight-bearing position.

Finally, the degree of dorsiflexion of the first MTPJ was recorded with the patient sitting in the resting position (first MTPJ ROM) and with the patient standing in a weight-bearing position (first MTPJ ROMw-b) (Figure 1). The center of the goniometer was placed on the center of the metatarsal head. The proximal arm was placed parallel to the floor, and the foot on the ground was held steady with one hand. The distal or mobile arm was placed parallel to the bisection of the proximal phalanx to avoid the influence of the interphalangeal joint ROM and held against the toe with the other hand. The maximum range of passive dorsiflexion was recorded [21].

Hallux rigidus was defined as a hallux dorsiflexion of less than 30° in a non-weight-bearing position. Functional hallux limitus was defined as the limitation of ROMw-b (<30°) of the first MTPJ in the absence of limitation of ROM (>40°) of the first MTPJ [8, 22].

### 2.3. X-Ray Goniometry

A weight-bearing lateromedial radiographic plane was obtained by using a standardized radiographic technique carried out by the same radiologist belonging to our department. Kodak Quality Control software POC 360 (Eastman Kodak Company, Rochester, NY) was used to calculate measurement angles.

A clinician different from the one who performed the clinical evaluation calculated the first metatarsal declination angle and was later blinded to any of the participants' clinical and personal data to avoid bias in the interpretation of the radiographic measurements. The following angles in the sagittal plane related to the ankle and the first MTPJ were calculated: calcaneal inclination angle, talar declination angle, talocalcaneal angle, tibiocalcaneal angle, tibiotalar angle, and first metatarsal declination angle [23].

### 2.4. Offloading Regime

All patients were off-loaded with therapeutic footwear and custom-made insoles and received an in-depth education on how to prevent ulceration. Therapeutic footwear consisting of off-the-shelf shoes with the following characteristics was prescribed: extradepth toe box, wide heel, laces or Velcro fasteners, seam-free inner lining, folds, and hollows. A list of therapeutic shoes was given to the patients in order to acquire them, and an experienced podiatrist evaluated fit and suitable characteristics. Custom insole was made from a positive plaster cast obtained from a neutral position of the foot. A metatarsal pad of Plastazote was placed behind the metatarsal heads. A cut-out refilled of Poron® was used to relieve local pressure, and a 45-degree shore hardness base of ethyl vinyl acetate (EVA) was used as the main structure of the insole. The dorsal cover was made of 25-degree shore hardness EVA. The total contact built into a multilayer construction with medial arch support replaced the removable standard insole of the shoe.

### 2.5. Follow-Up Protocol

All patients were followed-up prospectively until the development of an ulcer or until completion of the study (December 2017). Patients were evaluated every month according to the recommendations of the International Working Group on the Diabetic Foot [24]. At each monthly follow-up appointment, each patient was inspected for a new foot ulceration (according to the definition further below); if any callus/preulcerative lesions were observed, they were debrided, therapeutic footwear was monitored for effectiveness, and they were educated on the adherence to care.

Location of ulcer, foot type, presence of deformities, and joint mobility were recorded by the same clinician (RJMB).

### 2.6. Main Outcome Measure

Hallux reulceration during the follow-up period was assessed as the main outcome measure in the study. Hallux reulceration was defined as a new full-thickness lesion of the skin in the hallux, which is a new wound penetrating through the dermis of the hallux, without reference to time factors [25]. Potential factors responsible for casual pathogenesis of the new ulcers were evaluated according to the patient information and clinical findings.

### 2.7. Statistical Analysis

The assumption of normality of all continuous variables was verified using the Kolmogorov-Smirnov test. Normally distributed variables (Kolmogorov-Smirnov test with *p* ≥ 0.05) were reported as mean and standard deviations, and nonnormally distributed variables (Kolmogorov-Smirnov test with *p* < 0.05) were reported as medians and interquartile ranges.

Statistical differences in risk factors between patients who suffered hallux reulceration and those who did not were evaluated using the chi-square test for categorical variables, and the Student *t*-test was used for normally distributed quantitative variables.

The strength of difference in the effect size was calculated by the phi coefficient for the chi-square test and the *r* coefficient for the nonparametric test considering the values > 0.01 as a small effect, >0.30 as a medium effect, and >0.50 as a large effect. Cohen's *d* was calculated as the effect size for the parametric test using an effect size calculator (http://www.uccs.edu/~lbecker/) and considering the values > 0.2, >0.5, and>0.8 as small, moderate, and large effects, respectively. SPSS version 20.0 (SPSS, Chicago, IL, USA) was used for the other analyses.

The log-rank test was applied to determine the differences between risk factors in the time to hallux ulceration.

Continuous and categorical variables with *p* < 0.10 were selected as covariates in the univariate analysis to develop a Cox survival model of proportional hazards to determine the time to hallux ulceration and were expressed as a hazard ratio by using a forward stepwise selection method. *p* < 0.05 was accepted as statistically significant with a confidence interval of 95%. The Cox model excluded from the analysis those participants who had a shorter follow-up period than that conducted in the first event of hallux reulceration. A collinearity analysis was performed with the explanatory variables of the Cox model.

## 3. Results

Four patients dropped out of the study because of their refusal to wear protective therapeutic footwear and/or custom insole and were thus removed from the study. They presented no ulcers at the time of abandoning the study. Finally, 56 patients were included in the analysis. Patients were followed up prospectively for a median time period of 29 months (interquartile ranges (IR) 14.2-64.4 months).

Twenty-nine patients (52%) developed reulceration during follow-up and showed a median time to ulceration of 19 months (IR, 6.0-30.0 months). Different locations of reulceration were as follows: 9 patients (31%) developed a new ulcer in the hallux, 6 (21%) in the minor toes, 13 (45%) beneath the metatarsals, and 1 patient (3%) in the heel. All ulcers were classified as neuropathic reulceration, and there were no ulcers caused by trauma.

Several variables related to demographic and foot characteristics revealed differences between patients who developed hallux ulceration and patients who developed ulceration in other locations or without reulceration (Table 2). Participants who developed hallux ulceration were more likely to present a higher BMI (*p* = 0.030, Cohen's *d* = 0.404), a reduced dorsiflexion of the first MTFJ in a weight-bearing position (*p* = 0.041, Cohen's *d* = −0.422), and functional hallux limitus (*p* = 0.001, *r* = 0.428) as baseline characteristics. Hallux rigidus showed no association with hallux ulceration (*p* = 0.729, *r* = −0.046).

In the radiographic analysis (Table 2), lower first metatarsal inclination was associated with hallux ulceration (*p* = 0.024, Cohen's *d* = −0.350).

**Figure 2:**
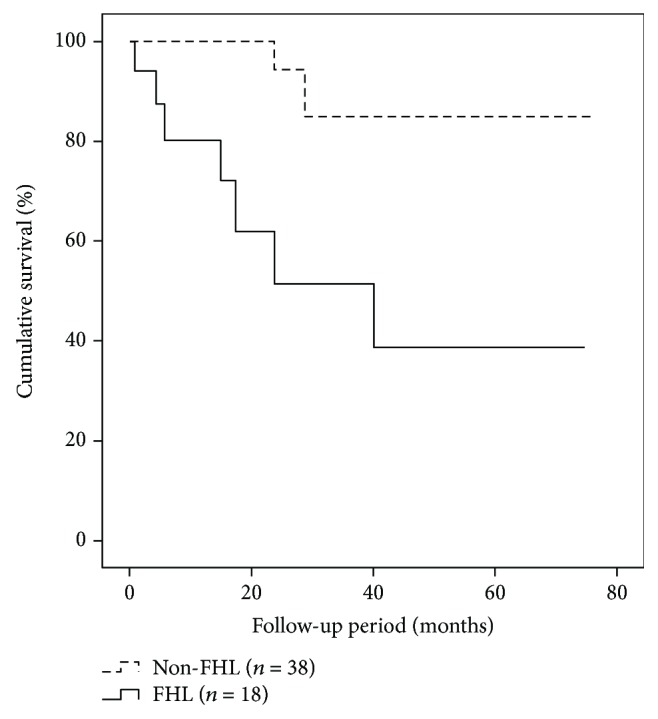
Kaplan-Meier analysis. Time to hallux reulceration for functional hallux limitus. Abbreviations: FHL: functional hallux limitus.

Figure 2 shows the association between functional hallux limitus and time to hallux ulceration (*p* = 0.027).

Functional hallux limitus and increased body mass index were the only variables associated with the time to hallux ulceration in the multivariate Cox model (*p* = 0.005, 95% CI (2.097–73.128), HR 12.384 and *p* = 0.044, 95% CI (1.003-1.272), HR 1.129, respectively).

## 4. Discussion

The findings of this study demonstrate that the presence of functional hallux limitus increases the probability of reulceration of the hallux in patients with a history of diabetic foot ulceration. The ROM of the first MTPJ is a determining factor for hallux pressures during midstance and propulsion, and it has been considered a probable cause of hallux ulceration [11]. However, to date, this relationship has not been demonstrated in patients with diabetes undergoing prospective follow-up.

One of the difficulties while debating on the limitation of ROM of the first MTPJ is the lack of consistency found within the literature relating to measurement of the joint [26]. Nubé et al. [14] evaluated the ROM of the hallux in the resting position in 60 patients with a history of previous foot ulcer and found no differences in the ROM between the hallux ulcer group and control ulcer group. In our study, an association with hallux reulceration was not found when hallux dorsiflexion was estimated in the non-weight-bearing position (*p* = 0.734, Cohen's *d* = −0.064).

Boffeli et al. [9] reported a high prevalence of limited ROM of the first MTPJ and functional hallux limitus in patients with previous hallux ulceration. However, they did not consider a control group, and therefore, statistical association was not investigated. Furthermore, a prospective follow-up of patients with previous hallux ulceration was not carried out.

Biomechanical theories about functional hallux limitus claim that both the elevation of the head of the first metatarsal and the increase in tension in the plantar aponeurosis may alter the joint dynamics in the first MTPJ. Owing to the ground reaction forces that are exerted on the first metatarsal head during midstance and propulsion phases, dorsiflexion of the first ray occurs in some patients, which can lead to blockage of the first MTPJ [13]. In our radiological univariate analysis, an increased dorsiflexion of first metatarsal declination was observed in patients who had previously developed hallux reulceration, which should support this theory. The evaluation of hallux dorsiflexion in a non-weight-bearing position does not regenerate the evidence about the weight borne by the head of the first metatarsal, and the result is normal, despite the fact that the joint dorsiflexion is blocked.

According to our results, we recommend to evaluate dorsiflexion of the hallux in the weight-bearing position and to then identify functional hallux limitus in the biomechanical screening of patients at high risk of foot reulceration.

Factors that contribute to a dorsiflexed first ray remain unknown [26]. An increased BMI value (*p* = 0.044; HR 1.129) may contribute, yet this is uncertain. A greater body weight has been previously found to be an independent risk factor for foot ulceration in a prospective study on a wide sample of patients with DM [27]. The presence of a high BMI and functional hallux limitus could predict the reulceration beneath the hallux, and we suggest that preventive strategies should be focused on the control of these variables.

Even though some authors have evaluated risk factors for hallux ulceration, this is the first study that prospectively explores functional hallux limitus as a risk factor for great toe reulceration, which represents the main strength of the study. However, our results should be interpreted with caution due to a number of limitations. Cumulative tissue stress has been shown to affect recurrence rates on the foot, and it is the result of the combination of plantar pressure and ambulatory activity [28]. However, neither of the variables was evaluated in this study. Secondly, the level of compliance of the patients was not evaluated in this study; the authors encouraged patients in each monthly visit to use therapeutic footwear, and all participants claimed to use preventive strategies. However, objective methods to evaluate compliance should be used in further studies [29].

## 5. Conclusions

In conclusion, the present study underscores the importance of identifying functional hallux limitus and demonstrates that patients with a history of DFU with functional hallux limitus and increased BMI have a higher probability of developing reulceration of the hallux.

## Figures and Tables

**Table 1:** Demographic data of the sample.

(*N* = 60)		Patients
Male/female		51 (85)/9 (15)
Mean age (years)		62 ± 8.3
Diabetes mellitus (years)		17 ± 13
Diabetes mellitus type 1/type 2		14 (23)/46 (77)
Ankle brachial index		1.18 ± 0.28
Retinopathy		31 (52%)
Nephropathy		15 (25%)
Body mass index (kg/m^2^)		29.4 (±5.4)
Location of the previous ulceration	Hallux	21 (35)
	Lesser toes	13 (22)
	Metatarsals	26 (43)
Glycated haemoglobin (mmol/mol) (%)		58 ± 9 (7.4 ± 1.2)

*n* (%) for categorical variables; mean ± SD for continuous variables.

**Table 2:** Differences between the risk factors for hallux reulceration.

(*N* = 56 patients)	Hallux reulceration (*n* = 9)	Nonhallux reulceration (*n* = 47)	*p* value	Effect size
Male/female	8 (88.9)/1 (11)	39 (83)/8 (17)	0.658	−0.059^a^
Mean age (years)	63 ± 9.7	62 ± 7.7	0.712	0.057^b^
Duration of DM (years)	9 ± 10.8	17 ± 13.3	0.072	−0.314^b^
Type 1/type 2 DM	0 (0)/9 (100)	13 (28)/34 (72)	0.072	0.241^a^
Body mass index (kg/m^2^)	33.6 ± 5.0	29.1 ± 5.2	0.030^∗^	0.404^b^
Nephropathy	3 (33)	12 (25)	0.628	0.065^a^
Retinopathy	4 (44)	25 (53)	0.630	−0.064^a^
HbA1c (mmol/mol) (%)	54 ± 6 (7.1 ± 0.8)	57 ± 8 (7.3 ± 1.1)	0.599	−0.207^b^
Ankle brachial index	1.43 ± 0.46	1.24 ± 0.28	0.254	0.242^b^
Hallux deformity	3 (33)	11 (23)	0.529	0.084^a^
Hallux valgus	1	10		
Metatarsal prominence	0	8		
Hallux hammertoe	2	2		
First MTPJ ROM (degrees)	47.33 ± 19.36	49.79 ± 19.15	0.734	−0.064^b^
First MTPJ ROM_w-b_ (degrees)	21.11 ± 7.15	33.26 ± 16.99	0.041^∗^	−0.422^b^
Functional hallux limitus	7 (78)	11 (23)	0.001^∗^	0.428^a^
Hallux rigidus	4 (44)	18 (38)	0.729	0.046^a^
Ankle ROM (degrees)	90.89 ± 5.49	87.60 ± 5.83	0.129	0.279^b^
Inversion ROM (degrees)	17.56 ± 4.87	16.85 ± 4.64	0.697	0.074^b^
Eversion ROM (degrees)	9.33 ± 1.41	9.74 ± 3.16	0.411	−0.083^b^
FPI	2.33 ± 2.50	0.66 ± 4.30	0.125	0.230^b^
Tibiotalar angle X-ray	112.01 ± 5.11	111.77 ± 6.21	0.902	0.021^b^
Tibiocalcaneal angle X-ray	67.96 ± 6.94	65.88 ± 7.44	0.433	0.143^b^
Talocalcaneal angle X-ray	45.11 ± 3.25	46.31 ± 6.13	0.403	−0.121^b^
Talar declination angle X-ray	26.64 ± 4.02	25.35 ± 4.16	0.397	0.156^b^
Calcaneal inclination angle X-ray	17.91 ± 4.51	20.91 ± 6.70	0.114	−0.254^b^
First metatarsal declination angle X-ray	22.81 ± 2.14	24.98 ± 3.50	0.024^∗^	−0.350^b^

Abbreviations: HbA1c: glycated hemoglobin; ROM: range of motion; first MTPJ ROM: range of dorsiflexion of the first metatarsophalangeal joint in the resting position; first MTPJ ROM_w-b_: range of dorsiflexion of the first metatarsophalangeal joints in the weight-bearing position; FPI: Foot Posture Index. The “nonhallux reulceration” group of patients included other locations of reulceration and patients who did not develop a new ulcer during the follow-up. ^a^*n* (%) for categorical variables; the phi coefficient was used for the chi-square test: representing effect size values of 0.01 as small effect, 0.30 as medium effect, and 0.50 as large effect. ^b^Mean ± SD for normally distributed variables; for independent samples, Student's *t*-test; effect size as the Cohen's *d*: representing effect size values > 0.2 as small effect, >0.5 as moderate effect, and >0.8 as large effect; *d* is positive if the mean difference is in the predicted direction.

## Data Availability

The datasets used to support this study are not freely available in view of participants' privacy protection.
